# The association of polypharmacy with intrinsic capacity: an analysis of the WHO ICOPE pilot data from Lianyungang, China

**DOI:** 10.3389/fmed.2025.1673885

**Published:** 2025-12-05

**Authors:** Xia Yun, Jing Wang, Ai Xia Guo, Zhi Ju Gu, Qian Sun, Jia Ming Xia, Qiong Jiang, Yuan Zhang, Yan Dong

**Affiliations:** 1 Department of Geriatrics, The Second Hospital of Shandong University, Shandong University, Jinan, Shandong, China; 2 The First People’s Hospital of Lanzhou City, Lanzhou, Gansu, China; 3 Lianyungang City Eastern Hospital, Lianyungang, Jiangsu, China; 4 First People’s Hospital of Lianyungang, Lianyungang, Jiangsu, China; 5 Guanyun County Traditional Chinese Medicine Hospital, Lianyungang, Jiangsu, China; 6 Haizhou District Puxi Community Health Service Center, Lianyungang, Jiangsu, China

**Keywords:** polypharmacy, intrinsic capacity (IC), older adults, ICOPE, integrated care

## Abstract

**Objective:**

As a core indicator of healthy aging, intrinsic capacity (IC) may be adversely impacted by polypharmacy. Leveraging data from the WHO Integrated Care for Older People (ICOPE) pilot in China, this study examines the association between polypharmacy and IC decline in older adults, aiming to provide evidence-based insights for optimizing geriatric pharmacotherapy.

**Methods:**

A cross-sectional analysis was conducted using data from community-dwelling and institutionalized adults aged ≥60 years enrolled at the Lianyungang (LYG) pilot site of the WHO ICOPE China program. Medication histories, demographic characteristics, lifestyle factors, and chronic disease profiles were collected via structured questionnaires. The WHO ICOPE screening tool was employed to assess five IC domains: cognition, mobility, sensory function, nutrition, and psychology. Polypharmacy was defined as concurrent use of ≥5 medications. Multivariable logistic regression models evaluated the polypharmacy-IC decline association, with stratification analyses assessing subgroup heterogeneity.

**Results:**

The study enrolled 467 participants, comprising a polypharmacy cohort (≥5 medications, *n* = 33) and a non-polypharmacy cohort (*n* = 434). Mean IC score was 3.5 ± 1.5. In unadjusted analyses, polypharmacy is associated with greater odds of IC decline (OR = 2.81, 95% CI: 1.06–7.43, *p* = 0.037). This association persisted in Model 1 (demographic-adjusted: OR = 2.82, 95% CI: 1.05–7.56, *p* = 0.039), Model 2 (socioeconomic-adjusted: OR = 3.65, 95% CI: 1.33–9.98, *p* = 0.012), and Model 3 (functional/geriatric-adjusted: OR = 3.23, 95% CI: 1.13–9.28, *p* = 0.029). However, in the fully adjusted Model 4 (including comorbidities), the OR attenuated to 2.31 (95% CI: 0.77–6.88, *p* = 0.134), retaining a positive but non-significant association. After full covariate adjustment, each additional medication was associated with a 22% increase in the likelihood of IC decline (OR = 1.22, 95% CI: 1.01–1.47, *p* = 0.04). Patients with IC decline demonstrated significantly higher medication counts than those with preserved IC (*p* < 0.05). Stratified analyses confirmed stable associations across subgroups (all interaction *p* > 0.05).

**Conclusion:**

An increase in the number of medications used by older adults may be positively associated with a decline in intrinsic capacity (IC). The prevalence of IC decline is significantly higher among individuals taking ≥5 medications compared to those using fewer medications. These findings suggest that polypharmacy is associated with IC decline and could serve as a potential indicator for IC decline. Prospective studies are needed to validate the causal relationship.

## 1 Introduction

The acceleration of global population ageing has driven a paradigm shift in healthcare from disease-centred to function-centred models to address challenges of profound ageing. In its World Report on Ageing and Health, the World Health Organization (WHO) established “healthy ageing” as the development and maintenance of functional ability enabling wellbeing in older age. Within this framework, WHO introduced the innovative construct of “intrinsic capacity” (IC)—defined as the composite of an individual’s physical and mental attributes—encompassing five domains: cognition, mobility, psychological, sensory (vision and hearing), and vitality (1), this conceptual advance signifies a critical transition from pathology-focused to function-oriented care (2). Complementing this, WHO has issued the Integrated Care for Older People (ICOPE) Guidelines, comprising the Manual: Person-Centered Assessment and Primary Care Pathway Guidelines, which establish standards for community interventions and clinical practice (3).

Over the past two decades, polypharmacy prevalence among older adults has risen substantially, emerging as a critical global public health challenge. The World Health Organization (WHO) defines polypharmacy as the concurrent use of five or more medications (4), evidence indicates that adverse drug reaction (ADR) incidence increases from 6% in older adults using two medications to 50% with five medications, approaching 100% when eight or more are administered (5). Among community-dwelling older adults, polypharmacy prevalence ranges from 4 to 86.6% (6), while in China, 66.4–75.3% exhibit impaired intrinsic capacity (7). These phenomena are mechanistically linked: approximately 69.1% of Chinese older adults experience decline in at least one intrinsic capacity domain (8), heightening vulnerability to polypharmacy. Notably, diminished intrinsic capacity constitutes a risk factor for potentially inappropriate medication (PIM) use in hospitalised elderly patients (9), IC decline and polypharmacy can accelerate functional decline, affect quality of life, and increase healthcare burden.

Polypharmacy poses a significant threat to the intrinsic capacity (IC) of older adults. This risk is primarily driven by the convergence of age-related pharmacokinetic/pharmacodynamic (PK/PD) alterations (e.g., reduced clearance, altered drug distribution), drug–drug interactions (DDIs), and cumulative drug toxicities (e.g., anticholinergic/sedative burden). These mechanisms collectively heighten vulnerability to adverse drug reactions and functional impairment across multiple IC domains. Existing evidence has established associations between polypharmacy and specific health impairments, including functional decline (10), cognitive deficits (11), elevated fall risk (12), and adverse drug events (13). Nevertheless, the holistic impact of polypharmacy on IC—mediated through the aforementioned synergistic mechanisms, particularly the dose–response relationship—remains inadequately characterized. To address this knowledge gap, we utilized data from the WHO ICOPE pilot program in Lianyungang, China. We aimed to investigate the association between polypharmacy and comprehensive intrinsic capacity (IC) decline using the WHO ICOPE integrated assessment framework.

## 2 Methods

### 2.1 Data and population

**Figure 1:**
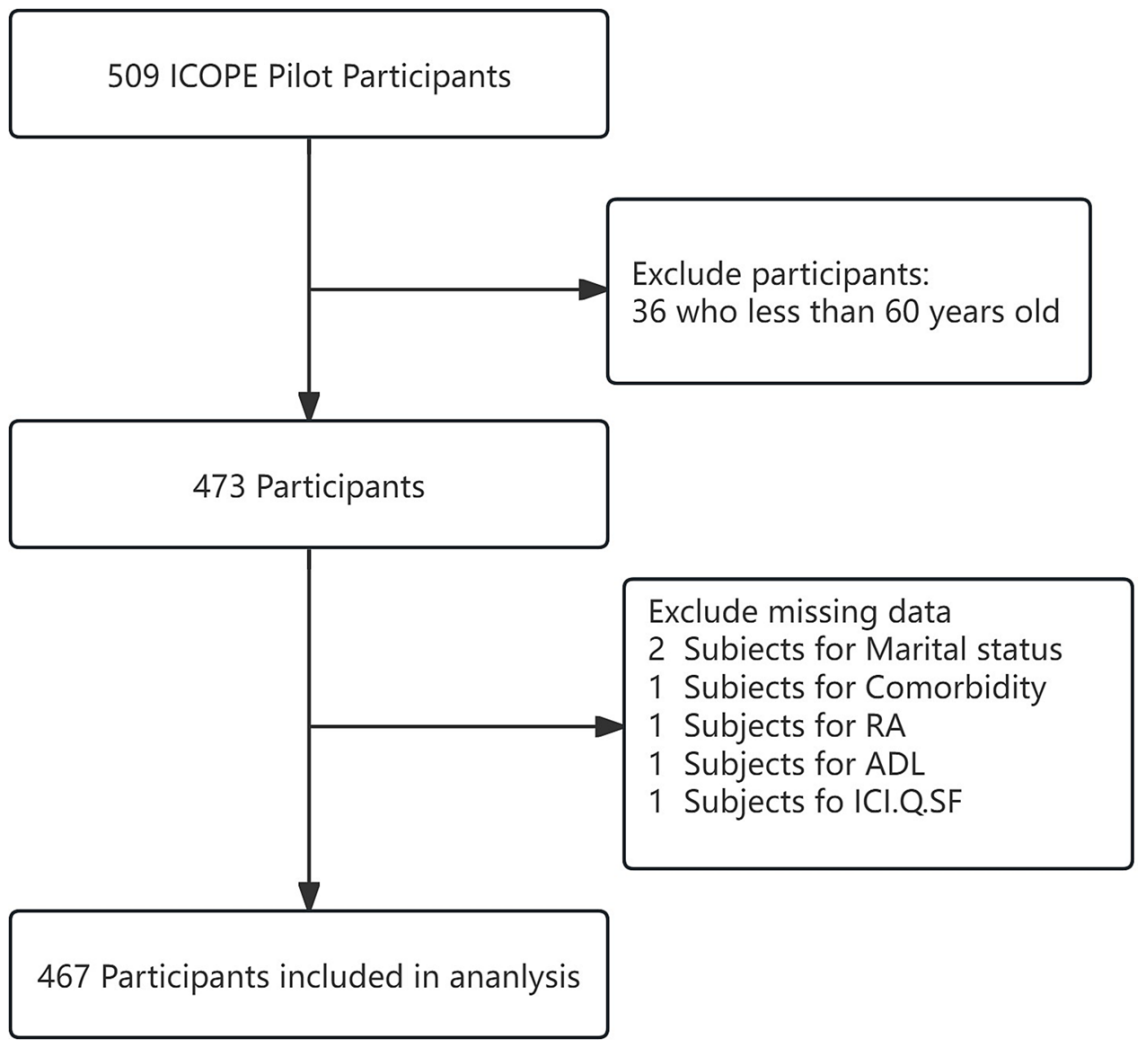
Flow chart of the study population.

This cross-sectional analysis utilised data from the WHO Integrated Care for Older People (ICOPE) pilot in Lianyungang City. Between March and April 2024, we enrolled adults aged ≥60 years residing locally for ≥6 months (community-dwelling and institutionalised populations) via convenience sampling at community health centres and long-term care facilities. Inclusion required: (1) baseline communication capacity for assessment completion; and (2) family member consent for participation support. Exclusion criteria comprised: (1) acute conditions necessitating urgent intervention; (2) incomplete baseline data; or (3) terminal illness with <6-month life expectancy. Of 509 screened participants, 467 met eligibility criteria and were analysed (Figure 1).

This study protocol was reviewed and approved by the Ethics Committee of Lianyungang First People’s Hospital on December 9, 2022, with ethics number QT-20221118001-02. The study complied with STROBE (Strengthening the Reporting of Observational Studies in Epidemiology) guidelines. Trained researchers administered pretested structured questionnaires using validated instruments. Rigorous quality control measures included: dual independent data entry, logic consistency validation, and outlier detection protocols throughout data collection.

### 2.2 Intrinsic capacity

Intrinsic capacity (IC) was assessed using the WHO ICOPE screening tool (3), comprising six domains: cognition, mobility, nutrition, vision, hearing, and psychological status. The reliability and validity of the tool have been well verified (14). Participants with positive initial screening results underwent further clinical evaluation. Cognition was quantified via the Mini-Mental State Examination (MMSE; score range 0–30), with impairment thresholds adjusted for educational attainment: ≤17 for illiterate individuals, ≤20 for primary school education, ≤22 for middle school, and ≤23 for university-level education.

Mobility assessment employed the Short Physical Performance Battery (SPPB), where scores ≤9 indicated impairment. Nutritional status was evaluated using the Mini Nutritional Assessment-Short Form (MNA-SF), defining malnutrition as MNA-SF scores ≤11 (15).

Those with positive results of visual acuity and hearing were further evaluated by comprehensive geriatric assessment tools commonly used by famous medical institutions in China (e.g., Peking Union Medical College Hospital and Beijing Geriatric Hospital). In-depth evaluation of visual acuity included the following: difficulty in reading/walking/watching TV, or occlusion or defect of vision, distortion of vision. A “no” score of 0 (good vision) was assigned for all three items, a “yes” score of 1 (poor vision) was assigned for 1–2 items, and a “yes” score of 2 (bad vision) was assigned for all three items. An in-depth hearing assessment includes the following questions: complaints about the volume of the television, the need for repetition, and difficulty listening to the phone. A “no” score for all three items was 0 (good hearing), a “yes” score for 1–2 items was 1 point (poor hearing), and a “yes” score for all three items was 2 points (bad hearing) (16).

According to the ICOPE guidelines (WHO, 2019) (17), the Psychological capacity dimension focuses on managing depressive symptoms. Its screening criteria are clear: If an individual has experienced any of the symptoms of “feeling down,” “feeling depressed,” “feeling hopeless” or “lack of interest/pleasure” in the past two weeks, they can be defined as having depressive symptoms.

Calculation of composite IC score: A composite IC score (range: 0–8) was derived by summing the scores assigned to each of the six domains. Cognition, mobility, nutrition, and psychological status (depressive symptoms) were scored dichotomously (0 = no impairment, 1 = impairment). Vision and hearing were scored on a three-level scale (0 = good, 1 = moderate impairment, 2 = severe impairment). IC decline (DIC) was defined as a composite score ≥ 2, while scores <2 denoted normal IC (non-DIC).

### 2.3 Polypharmacy

Polypharmacy is defined as the prescription of five or more different medications taken daily. The types of medications included in polypharmacy encompass both prescription and over-the-counter drugs (18), while herbal remedies and supplements were excluded. During the baseline survey, the number of medications taken by patients was assessed by asking, “What medications have you been taking daily for the past three months?” to gather information on their recent medication use.

### 2.4 Covariates

Key sociodemographic and clinical covariates were adjusted for in the analyses. Sociodemographic factors encompassed: age (<80 vs. ≥80 years), marital status (married/unmarried), educational attainment (illiterate/primary school vs. ≥junior high school), residential setting (urban/suburban), nursing home residence (yes/no), and caregiver availability (yes/no). Clinical covariates incorporated frailty status, social functioning (assessed via Social Participation Frequency, SPF), basic activities of daily living (ADL), urinary incontinence (UI), and Charlson comorbidity index (CCI) scores.

Frailty was assessed using the K-FRAIL scale (19), comprising five criteria: fatigue, resistance, ambulation, illnesses, and weight loss (20). Each criterion was scored dichotomously (0/1), with total scores categorised as: robust (0), pre-frail (1–2), or frail (≥3).

Social Participation Function (SPF) was evaluated through a validated instrument assessing five domains (21): appropriate social adaptation (score 0), adaptation to simple environments with undetectable cognitive issues at initial contact (score 1), social withdrawal with passive interaction, impaired initiation, and inappropriate remarks increasing deception vulnerability (score 2), limited interactions with unclear speech/inappropriate expressions (score 3), markedly impaired social engagement (score 4), total SPF scores defined functional levels: intact (0–2), mild (3–7), moderate (8–13), or severe impairment (14–20).

Activities of daily living (ADL) were measured using the modified Barthel index (MBI; range 0–100), classifying dependency as: complete independence (100), mild (91–99), moderate (61–90), severe (21–60), or total dependence (≤20).

The International Consultation on Incontinence Questionnaire-Urinary Incontinence Short Form (ICIQ-UI SF) assesses the severity of urinary incontinence (UI) symptoms. It comprises three questions: Question 1 quantifies the frequency of urine leakage, Question 2 evaluates the amount of leakage, and Question 3 measures the impact of UI on daily life. The total ICIQ score ranges from 0 to 21. A score of 0 indicates no incontinence; 1–5: mild incontinence; 6–12: moderate incontinence; 13–21: severe incontinence (22).

Comorbidity burden was quantified using the Charlson comorbidity index and Cumulative Illness Rating Scale-Geriatrics (CIRS-G), supplemented by clinical expertise. Seventeen disease categories (e.g., myocardial infarction, chronic pulmonary disease, malignancies) were graded 1–6 based on severity. Total scores ≥1 indicated comorbidity presence, with higher scores reflecting greater burden (0 = no comorbidity) (23).

### 2.5 Statistical analysis

Descriptive statistics characterised baseline participant characteristics: normally distributed continuous variables as mean ± standard deviation (SD), non-normally distributed variables as median (interquartile range, IQR), and categorical variables as frequency (percentage). Group comparisons used independent samples *t*-tests or Mann–Whitney *U* tests for continuous variables, and *χ*^2^ or Fisher’s exact tests for categorical variables.

Multivariable logistic regression examined polypharmacy (≥5 medications)-intrinsic capacity (IC) associations. To address confounding and validate robustness, covariates were selected through: univariate analysis with *p* < 0.05 (Supplementary Table 1); ≥10% change-in-estimate criterion; clinically relevant variables per expert consensus. Four adjusted models generated odds ratios (ORs) with 95% CIs. Subgroup analyses assessed associations across strata. We employed the nonparametric Kruskal–Wallis *H* test to compare differences in medication counts among the high-, medium-, and low-level intrinsic capability (IC) groups. Given minimal missing data (0–6%), case deletion was applied. All analyses were conducted using the statistical software packages R (http://www.r-project.org, The R Foundation) and Free Statistics software versions, with a two-sided *p*-value <0.05 considered statistically significant.

## 3 Results

### 3.1 Patient characteristics

The cohort comprised 467 older adults (Figure 1), stratified into non-polypharmacy (<5 medications, *n* = 434) and polypharmacy (≥5 medications, *n* = 33) groups. The prevalence of DIC was 67.9% (317/467). Compared with the non-polypharmacy group, the polypharmacy group demonstrated significantly higher proportions of comorbidities, frailty, urban residency, vision disorder and married status (*p* < 0.05; Table 1). IC scores were marginally elevated in polypharmacy users (mean ± SD: 4.0 ± 1.5 vs. 3.5 ± 1.5; *p* = 0.054), with significantly increased incidence of IC decline (84.8% vs. 66.6%; *p* = 0.03). No significant intergroup differences were observed in: sex distribution, age stratification, BMI, Mini-Mental State Examination (MMSE) scores, malnutrition, depression, SPF, sleep quality, and ADL levels (all *p* > 0.05).

**Table 1:** Characteristics of the older participants.

Variables	Total (*n* = 467)	Number of medications		*p*
		<5 (*n* = 434)	≥5 (*n* = 33)	
IC, mean ± SD	3.5 ± 1.5	3.5 ± 1.5	4.0 ± 1.5	0.054
Sex, *n* (%)				0.21
Male	206 (44.1)	188 (43.3)	18 (54.5)	
Female	261 (55.9)	246 (56.7)	15 (45.5)	
Age, *n* (%)				0.208
<80 years	190 (40.7)	180 (41.5)	10 (30.3)	
≥80 years	277 (59.3)	254 (58.5)	23 (69.7)	
BMI (kg/m^2^) mean ± SD	25.0 ± 5.6	25.0 ± 5.5	25.3 ± 6.7	0.742
Comorbidity, median (IQR)	1.0 (0.0, 3.0)	1.0 (0.0, 2.0)	4.0 (1.0, 7.0)	< 0.001
ICIQ-SF, *n* (%)				0.056
No	361 (77.3)	340 (78.3)	21 (63.6)	
Mild	48 (10.3)	40 (9.2)	8 (24.2)	
Moderate	34 (7.3)	31 (7.1)	3 (9.1)	
Severe	24 (5.1)	23 (5.3)	1 (3)	
Frail, *n* (%)				0.001
No	380 (81.4)	360 (82.9)	20 (60.6)	
Yes	87 (18.6)	74 (17.1)	13 (39.4)	
Education, *n* (%)				0.07
Illiterate	158 (33.8)	150 (34.6)	8 (24.2)	
Primary school	141 (30.2)	134 (30.9)	7 (21.2)	
Junior high school and above	168 (36.0)	150 (34.6)	18 (54.5)	
Residential area (RA), *n* (%)				0.004
Suburban	163 (34.9)	159 (36.6)	4 (12.1)	
Urban	304 (65.1)	275 (63.4)	29 (87.9)	
Marital status, *n* (%)				0.045
Married	247 (52.9)	224 (51.6)	23 (69.7)	
Unmarried	220 (47.1)	210 (48.4)	10 (30.3)	
Nursing home, *n* (%)				0.247
No	252 (54.0)	231 (53.2)	21 (63.6)	
Yes	215 (46.0)	203 (46.8)	12 (36.4)	
MMSE, *n* (%)				0.245
No	341 (73.2)	314 (72.5)	27 (81.8)	
Yes	125 (26.8)	119 (27.5)	6 (18.2)	
Malnutrition, *n* (%)				0.109
No	403 (86.3)	378 (87.1)	25 (75.8)	
Yes	64 (13.7)	56 (12.9)	8 (24.2)	
Depression, *n* (%)				0.111
No	17 (3.6)	14 (3.2)	3 (9.1)	
Yes	450 (96.4)	420 (96.8)	30 (90.9)	
SPPB, *n* (%)				0.075
No	355 (76.3)	334 (77.3)	21 (63.6)	
Yes	110 (23.7)	98 (22.7)	12 (36.4)	
Vision, *n* (%)				0.001
Good	333 (71.3)	319 (73.5)	14 (42.4)	
Moderate	94 (20.1)	80 (18.4)	14 (42.4)	
Poor	40 (8.6)	35 (8.1)	5 (15.2)	
Hearing, *n* (%)				0.891
Good	310 (66.4)	289 (66.6)	21 (63.6)	
Moderate	52 (11.1)	48 (11.1)	4 (12.1)	
Poor	105 (22.5)	97 (22.4)	8 (24.2)	
SPF, *n* (%)				0.159
No impairments	382 (81.8)	352 (81.1)	30 (90.9)	
Impairments	85 (18.2)	82 (18.9)	3 (9.1)	
Sleep, *n* (%)				0.213
No impairments	390 (83.5)	365 (84.1)	25 (75.8)	
Impairments	77 (16.5)	69 (15.9)	8 (24.2)	
ADL, *n* (%)				0.15
High level	334 (71.5)	314 (72.4)	20 (60.6)	
Low level	133 (28.5)	120 (27.6)	13 (39.4)	
DIC, *n* (%)				0.03
No	150 (32.1)	145 (33.4)	5 (15.2)	
Yes	317 (67.9)	289 (66.6)	28 (84.8)	

IC, intrinsic capacity; DIC, decreased intrinsic capacity; BMI, body mass index; ICIQ-SF, International Consultation on Incontinence Questionnaire-Short Form; RA, residential area; MMSE, Mini-Mental State Examination; MNA-SF, Mini Nutritional Assessment-Short Form; SPF, Social Participation Function; ADL, activities of daily living.

### 3.2 The association between polypharmacy and intrinsic capacity

Table 2 presents the multivariable regression analyses, which indicate a positive association between the number of medications and decline in intrinsic capacity (IC). When treated as a continuous variable, each additional medication was associated with greater odds of IC decline in the crude model (OR = 1.29, 95% CI: 1.10–1.52, *p* = 0.002). After sequential adjustment for demographic characteristics (Model 1: OR = 1.28, 95% CI: 1.09–1.51, *p* = 0.003), socioeconomic factors (Model 2: OR = 1.36, 95% CI: 1.15–1.62, *p* < 0.001), functional status indicators (Model 3: OR = 1.30, 95% CI: 1.08–1.55, *p* = 0.005), and comorbidities (Model 4: OR = 1.22, 95% CI: 1.01–1.47, *p* = 0.04), these associations remained statistically significant.

**Table 2:** Multivariable logistic regression analysis of the association between intrinsic capacity and polypharmacy.

Variable	*N* = 467	Crude model		Model 1		Model 2		Model 3		Model 4	
		OR (95% CI)	*p*-value	OR (95% CI)	*p*-value	OR (95% CI)	*p*-value	OR (95% CI)	*p*-value	OR (95% CI)	*p*-value
Number of medications	467	1.29 (1.09–1.51)	0.002	1.28 (1.09–1.51)	0.003	1.36 (1.15–1.62)	<0.001	1.3 (1.08–1.55)	0.005	1.22 (1.01–1.47)	0.04
Number of medications											
<5	434	1 (Ref)		1 (Ref)		1 (Ref)		1 (Ref)		1 (Ref)	
≥5	33	2.81 (1.06–7.43)	0.037	2.82 (1.05–7.56)	0.039	3.65 (1.33–9.98)	0.012	3.23 (1.13–9.28)	0.029	2.31 (0.77–6.88)	0.134

OR, odds ratio; CI, confidence interval.

Crude model: unadjusted.

Model 1: Gender, age, BMI.

Model 2: Gender, age, BMI, marital, residential area, education.

Model 3: Gender, age, BMI, marital, residential area, education, nursing home, ADL, SPF, ICIQ-SF, frail.

Model 4: Gender, age, BMI, marital, residential area, education, nursing home, ADL, SPF, ICIQ-SF, frail, comorbidity.

When stratified by medication threshold (with <5 medications as the reference group), polypharmacy (≥5 medications) was significantly associated with a decline in intrinsic capacity (IC) in the unadjusted model (OR = 2.81, 95% CI: 1.06–7.43, *p* = 0.037). This association was maintained in Model 1 (OR = 2.82, 95% CI: 1.05–7.56, *p* = 0.039) and further amplified in Model 2 (OR = 3.65, 95% CI: 1.33–9.98, *p* = 0.012). However, in Model 4, after adjusting for comorbidities, the odds ratio decreased to 2.31 (95% CI: 0.77–6.88, *p* = 0.134), no longer reaching statistical significance.

In Supplementary Table 2, we demonstrated a significant association between medication burden and vision impairment. Each additional medication increased the odds of vision impairment by 43% (OR = 1.43, 95% CI: 1.24–1.63, *p* < 0.001). Likewise, polypharmacy was significantly associated with elevated odds of vision impairment (OR = 3.76, 95% CI: 1.83–7.75, *p* < 0.001). Concurrently, a positive trend was observed between increasing medication count and impairments in locomotion, nutritional risk, and hearing, although these associations failed to reach statistical significance.

**Figure 2:**
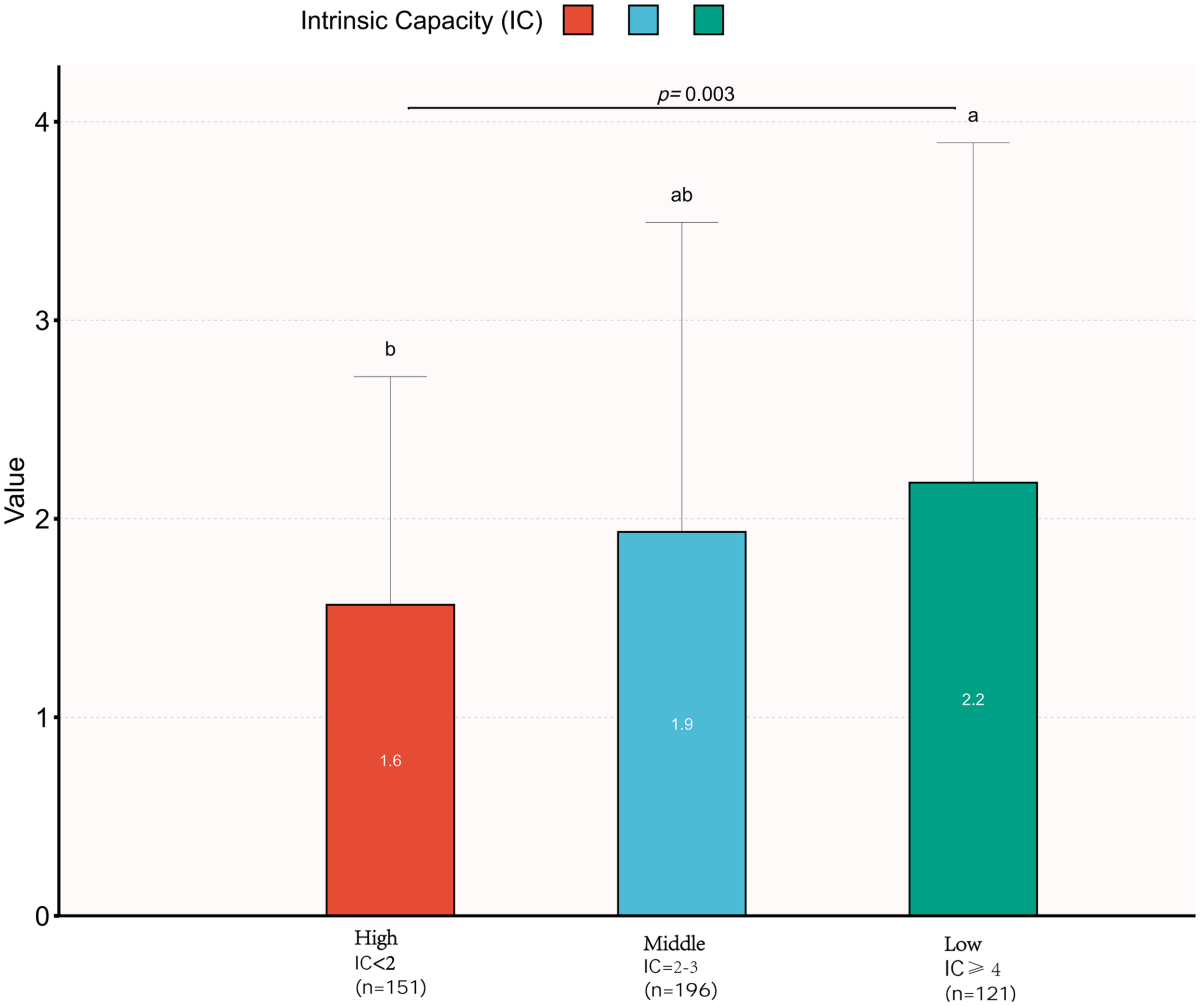
Comparison of number of medications across intrinsic capability (IC) levels. A statistically significant difference in medication counts was observed among IC-level groups (Kruskal–Wallis *H* test: *H* = 12.801). Groups marked with different superscript letters denote significant pairwise differences based on Dunn’s *post hoc* test with Bonferroni correction (*p* < 0.05).

Figure 2 demonstrates significant differences in the distribution of medication numbers among different intrinsic capacity (IC) level groups (Kruskal–Wallis *H* = 12.80, *p* < 0.05). The high IC group (<2 points) had the lowest median number of medications (1.6), followed by the medium IC group (2–3 points) with 1.9 medications, and the low IC group (≥4 points) had the highest (2.2 medications), demonstrating an increasing trend in medication numbers with decreasing IC levels.

### 3.3 Subgroup analyses

**Figure 3:**
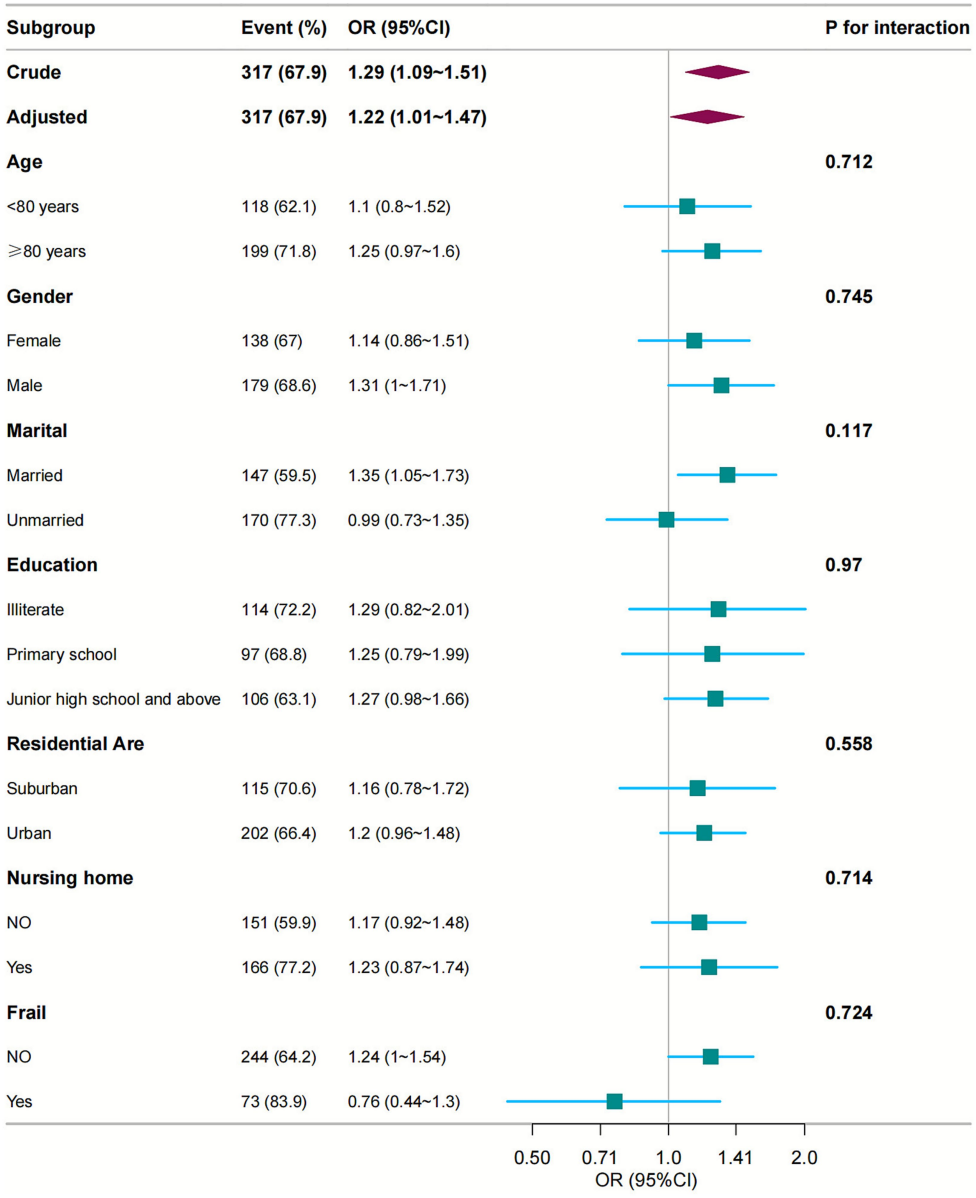
Subgroup analyses of the relationship between number of medications and intrinsic capacity. Odds ratios were adjusted for variables as in Model 4 (Table 2) except for the corresponding stratification variable. CI, confidence interval; OR, odds ratios.

We conducted subgroup analyses to evaluate potential effect modifiers in the medication count-diminished intrinsic capacity association. Participants were stratified by age (<80 vs. ≥80 years), sex (female/male), marital status (married/unmarried), education level, residential area, and institutional care status. Forest plots were generated to visualise trend consistency across strata. Notably, no significant interaction effects were detected for any subgroup variable (all interaction *p*-values >0.05; Figure 3).

## 4 Discussion

This study utilized data from the World Health Organization (WHO) Integrated Care for Older People (ICOPE) pilot program in China to investigate the association between polypharmacy and intrinsic capacity (IC) in older adults. The findings revealed that: each additional medication was associated with a 22% increased likelihood of decline in intrinsic capacity. Notably, polypharmacy (≥5 medications) was significantly associated with a higher prevalence of IC decline (84.8% vs. 66.6%). Older adults with lower IC levels had a higher average number of medications (see Figure 2). Subgroup analyses systematically evaluated effect modification across different demographic subgroups. The robust positive association between polypharmacy and IC impairment remained consistent across all subgroups (*p* for interaction >0.05), indicating significant population generalizability of this association. These observations are consistent with existing evidence. Previous studies have identified five distinct patterns of intrinsic capacity impairment, among which “depression with cognitive impairment” and “pan-domain impairment” are strongly associated with excessive polypharmacy (1), this indicates that polypharmacy exacerbates IC decline and increases the risk of adverse health outcomes (24).

Our investigation detected diminished intrinsic capacity (IC) in 67.9% of elderly participants, consistent with worldwide patterns. Chinese research involving 376 seniors recorded 69.1% exhibiting ≥1 affected IC domain (8). Parallel findings emerged from a WHO-associated French center, where the 9-item ICOPE screen revealed 92.6% IC impairment prevalence among 755 older adults (25). Cumulatively, these outcomes confirm compromised IC as a widespread geriatric condition. Significantly, research on Chinese subjects aged ≥50 years initially indicated poorer IC in polypharmacy recipients, although age adjustment nullified this correlation statistically (7). Such inconsistency potentially stems from age-medication collinearity: progressive aging typically heightens multimorbidity probability, thereby augmenting polypharmacy likelihood. To resolve this, we performed stratified age analyses (<80 versus ≥80 years). Outcomes verified markedly increased IC deterioration in octogenarians. Crucially, age-adjusted models maintained statistically significant polypharmacy-IC links, positioning medication overload as an autonomous predictor of IC deterioration irrespective of biological aging.

Progressive aging increases susceptibility to chronic disorders and geriatric syndromes in older adults, frequently requiring multidrug regimens for symptom control. For instance, a US meta-analysis documented significantly higher polypharmacy prevalence among individuals aged ≥65 years compared with younger populations (26). Indicating extensive medication burden in senescence. While polypharmacy inherently reflects comorbid disease presence, it may also directly compromise intrinsic capacity through pharmacological mechanisms. Notably, our analysis observed marked attenuation of the polypharmacy-intrinsic capacity association following comorbidity adjustment in Model 4. This attenuation likely reflects multicollinearity between medication burden and comorbidity measures. Although Yoshida et al. (10) detected no significant multimorbidity-polypharmacy interaction effect on physical function, polypharmacy remains plausible to accelerate functional deterioration through drug–drug interactions and drug-disease incompatibilities. Future research using longitudinal designs is needed to disentangle their independent effects on functional decline.

Beyond comorbidity influences, polypharmacy subgroups demonstrated higher proportions of metropolitan dwellers and married individuals. Urban populations typically exhibit enhanced health consciousness and financial capacity, compounded by greater healthcare resource availability in cities, collectively facilitating improved medical access. These determinants synergistically promote medication intensification among urban residents. Concurrently, marital status provides spousal engagement and socio-emotional support, potentially fostering proactive health monitoring and prompt healthcare-seeking behaviour (27). Consequently, urban residency and marital union emerge as salient contributing factors to polypharmacy patterns.

Studies indicate that polypharmacy is strongly associated with impaired intrinsic capacity (IC) and geriatric syndromes, including frailty, cognitive decline, and falls (28, 29). Evidence suggests a bidirectional relationship wherein polypharmacy exacerbates frailty and functional decline (30). While multimorbidity-driven therapeutic complexity perpetuates medication overload. Notably, regimens involving ≥5 medications are linked to significantly higher risks of frailty (2.5-fold) (31), mobility impairment, and mortality (29, 30), whereas limited regimens (1–4 drugs) may exert protective effects (10). Our analysis demonstrates a clinically significant association between pharmacological burden and visual impairment, with each additional medication conferring a 43% increased odds of vision loss. Notably, commonly prescribed drug classes—including anticholinergics, corticosteroids, and psychoactive agents—may induce cataract formation, glaucoma progression, or retinal toxicity (32). Vision loss functionally compromises activities of daily living (ADL) (33), thereby accelerating intrinsic capacity (IC) decline. Critically, as visual function constitutes a core IC component within the WHO Integrated Care for Older People (ICOPE) framework, its impairment directly diminishes global IC scores. These findings collectively underscore the detrimental impact of polypharmacy on physical function and intrinsic capacity (34, 35). The pathophysiological mechanisms underlying polypharmacy-induced IC impairment may stem from drug–drug interactions, cumulative adverse effects (e.g., anticholinergic burden) (36, 37), and age-related pharmacokinetic decline (38, 39). Though this complex relationship remains incompletely elucidated.

To mitigate the adverse effects of polypharmacy on the intrinsic capacity (IC) of older adults, we recommend establishing a structured, community-based, multidisciplinary management pathway (8). This pathway should actively engage physicians and nurses, clinical pharmacists, nutritionists, psychologists, and rehabilitation therapists by: implementing Electronic Health Record (EHR) alerts during clinical consultations to flag polypharmacy; integrating polypharmacy management into the training curricula of relevant healthcare professionals. We have identified polypharmacy as a significant and dose-dependent risk marker, making it a practical lever for intervention. During the initial stages of ICOPE screening, priority should be given to individuals taking five or more medications for comprehensive medication reviews and in-depth assessments, leading to the development of personalized care plans that include deprescribing strategies and medication optimization. This integrated approach, combining ICOPE integration, clearly defined multidisciplinary pathways, and targeted policy interventions, aims to optimize medication use and preserve intrinsic capacity in the older population.

### 4.1 Limitations

Methodological strengths of this study include its integration with the World Health Organization (WHO) Integrated Care for Older People (ICOPE) pilot program. By rigorously adhering to the ICOPE protocol and including both community-dwelling and nursing home residents, we established a dose–response relationship between the number of medications and intrinsic capacity (IC). However, the following limitations should be noted. The cross-sectional design precludes causal inference regarding the relationship between polypharmacy and IC. Due to the post-hoc nature of the subgroup analyses, the relatively small sample size in the polypharmacy subgroup limited the statistical power of some analyses, and the results warrant cautious interpretation. Potential multicollinearity among covariates (such as comorbidity burden) may have been present; the observed effect of polypharmacy was attenuated after adjusting for comorbidities in the multivariate regression analysis. Furthermore, reliance on self-reported medication data (susceptible to recall bias) and the lack of prescription details (e.g., drug classes, potentially inappropriate medications) may have influenced the findings. Although we adjusted for key covariates including age, comorbidities, and functional status, the potential impact of unmeasured confounders must be considered. Factors such as social support, lifestyle and diet, and medication adherence could concurrently influence both prescribing patterns and IC trajectories (40). Future research should employ multi-center, prospective, large-sample cohort designs, incorporate a broader range of variables, and track IC trajectories over time to further elucidate the causal relationship and underlying mechanisms between polypharmacy and intrinsic capacity.

## 5 Conclusion

In conclusion, based on the analysis of WHO ICOPE pilot data from China, increased medication burden appears associated with declining intrinsic capacity (IC) in older adults. Individuals with polypharmacy (≥5 medications) exhibited higher prevalence of IC deterioration, suggesting that polypharmacy may be associated with IC decline and could serve as an indicator for IC decline. These findings underscore the necessity of addressing polypharmacy risks in comprehensive care for older adults. Future validation studies should integrate medication categories with longitudinal tracking of intrinsic capacity functional trajectories.

## Data Availability

The data analyzed in this study is subject to the following licenses/restrictions: the datasets generated during this study are available from the corresponding author on reasonable request. Requests to access these datasets should be directed to YD, dylzu_lnyx@163.com.
