# Supplementary material for: The association of polypharmacy with intrinsic capacity: an analysis of the WHO ICOPE pilot data from Lianyungang, China
Source: Front Med (Lausanne). 2025 Dec 5;12:1673885. doi: 10.3389/fmed.2025.1673885 (PMC12715426; doi:10.3389/fmed.2025.1673885)
Supplement: Supplementary file 1 [file Data_Sheet_1.docx]

**Supplementary**

**Supplementary table 1.**Univariate Analysis of Decline in Intrinsic Capacity

| Variable | OR 95%CI | *P* value |
| --- | --- | --- |
| Female | 1.08 (0.73~1.59) | 0.714 |
| Age≥80 | 1.56 (1.05~2.31) | 0.027 |
| BMI(kg/m^2^） | 0.98 (0.95~1.02) | 0.307 |
| Comorbidity | 1.22 (1.09~1.37) | <0.001 |
| Frail | 2.91 (1.58~5.34) | 0.001 |
| Urban | 0.83 (0.55~1.25) | 0.365 |
| Unmarried | 2.31 (1.54~3.47) | <0.001 |
| Nursing.home | 2.27 (1.51~3.4) | <0.001 |
| MMSE | 30.81 (9.61~98.82) | <0.001 |
| Depression | 17.5 (3.95~77.58) | <0.001 |
| SPF | 6.67 (2.99~14.85) | <0.001 |
| Sleep | 4.28 (2.07~8.84) | <0.001 |
| SPPB | 77.03 (10.63~558.06) | <0.001 |
| ADL | 3.87 (2.27~6.6) | <0.001 |
| ICI.Q.SF | 3.62 (2.02~6.51) | <0.001 |

**Note:**BMI,body mass index;RA,Residential Area;MMSE,Mini-mental state examination;MNA-SF,Short-form mini-nutritional assessment;SPF，Social Participation Function; ADL,Activities of Daily Living,ICI.Q.SF,International Consultation on Incontinence Questionnaire-Short Form

| Variable | MMSE | | SPPB | | Malnutrition | | Vision | | Hearing | | Depression | |
| --- | --- | --- | --- | --- | --- | --- | --- | --- | --- | --- | --- | --- |
|  | OR（95CI） | *P* | OR （95CI） | *P* | OR （95CI） | *P* | OR （95CI） | *P* | OR（95CI） | *P* | OR（95CI） | *P* |
| Number of Medications | 1 (0.87~1.15) | 0.986 | 1.12 (0.98~1.28) | 0.101 | 1.15 (0.99~1.34) | 0.067 | 1.43 (1.24~1.63) | <0.001 | 1.13 (1~1.28) | 0.054 | 0.93 (0.7~1.24) | 0.617 |
| Number of Medications |  |  |  |  |  |  |  |  |  |  |  |  |
| ＜5 | 1(Ref) |  | 1(Ref) |  | 1(Ref) |  | 1(Ref) |  | 1(Ref) |  | 1(Ref) |  |
| ≥5 | 0.59 (0.24~1.46) | 0.25 | 1.95 (0.93~4.1) | 0.079 | 2.16 (0.93~5.02) | 0.074 | 3.76 (1.83~7.75) | <0.001 | 1.14 (0.55~2.38) | 0.729 | 0.33 (0.09~1.22) | 0.098 |

**Supplementary Table 2:** Univariate Analysis of Associations between Medication Count and Domain-Specific Impairments
